# Feasibility of video-assisted cardiopulmonary resuscitation when the lay responder is alone with the victim: a randomized controlled crossover pilot study

**DOI:** 10.1038/s41598-025-12014-6

**Published:** 2025-07-19

**Authors:** Bálint Bánfai, József Betlehem, Boróka Jobb, Balázs Horváth, Kitti Máté-Póhr, Dániel Németh, János Musch, Henrietta Bánfai-Csonka

**Affiliations:** 1 Faculty of Health Sciences, Institute of Emergency Care, Pedagogy of Health and Nursing Sciences, University of Pécs, Vörösmarty street 4, Pécs, 7621 Hungary; 2 Faculty of Health Sciences, Human Patient Simulation Center, University of Pécs, Szepesy street 1-3, Pécs, 7621 Hungary; 3 Faculty of Health Sciences, Doctoral School of Health Sciences, University of Pécs, Vörösmarty street 4, Pécs, 7621 Hungary; 4 National Ambulance Service, Budapest, Hungary

**Keywords:** Cardiology, Cardiovascular diseases, Health care, Health services, Patient education, Public health

## Abstract

Video-assisted cardiopulmonary resuscitation (V-CPR) can improve the quality of bystander-performed CPR; however, its applicability has not been explored in situations where the lay responder is alone with the victim. This pilot study aimed to assess the feasibility of V-CPR in such scenarios. A randomized controlled crossover simulation study was conducted with 10 lay responders in two settings: a residential living room and a yard. Participants used available household objects to position a smartphone for a live video call with a dispatcher. The study evaluated (1) camera placement and perspective, (2) camera placement time, and (3) video quality and CPR assessability, based on assessors evaluations. No participant failed to establish a usable camera view. The average placement time was 23.4 s indoors and 34.0 s outdoors (*p* = 0.075). Based on five assessors, hand position and compression rate were highly assessable (86% and 84%), while compression depth was correctly assessed in only 32%. Although V-CPR quality varied between environments, the method proved feasible even when responders acted alone. Further studies are needed to compare the results with commercial V-CPR, optimize camera positioning, reduce delays, and evaluate outcomes using standardized dispatcher protocols. This study was retrospectively registered at https://clinicaltrials.gov/ClinicalTrials.gov (NCT06794398, 01/27/2025).

**Supplementary Information:**

The online version contains supplementary material available at 10.1038/s41598-025-12014-6.

## Introduction

Out-of-hospital cardiac arrest (OHCA) is a global problem and the third leading cause of death in Europe^1^. In OHCA, the first links of the “chain of survival” (early recognition, calling the ambulance, and initiating effective cardiopulmonary resuscitation, CPR) by lay responders are essential before the ambulance arrives to increase survival rates and provide better neurological outcomes^1–3^.

Despite wider community actions in recent years, CPR rates initiated by lay responders remain lower than expected^1^. The implementation of new technologies for OHCA management is highly recommended^4^. The “Systems saving lives” approach appointed five key areas for improving survival rates^5–7^. One of these areas is the improvement of dispatcher-assisted cardiopulmonary resuscitation (CPR). Telephone-assisted CPR (T-CPR) is a feasible and effective way to help the caller identify cardiac arrest and promptly initiate CPR with the motivation and instructions of the dispatcher^5–7^. Neurologically intact survival at discharge and after one month also increased with dispatcher-assisted CPR^8^.

Video-assisted CPR (V-CPR) is a relatively new method that allows dispatchers to see the environment and the victim and can provide more precise instructions and feedback to lay responders than T-CPR^9–12^. Recent studies have shown better clinical outcomes and more effective chest compressions when using V-CPR compared to unassisted CPR or T-CPR^13–15^. At the same time, some technical issues can influence the feasibility and effectiveness of V-CPR (e.g., camera position, quality of the picture, lack of signal, environmental factors such as too bright light or darkness, bad weather)^10,16,17^.

Furthermore, V-CPR is available only when at least two lay responders are present (one provides CPR and the other holds the camera). Most previous simulation studies used a previously installed tripod or involved a study operator to reach the appropriate camera perspective; in real situations, a second responder held the camera^10^. However, a second person may not always be present during a real emergency.

Therefore, recommendations are essential to provide a quick and evaluable smartphone camera perspective even when the lay responder is alone with the OHCA patient. To the best of our knowledge, no previous study has examined whether a self-installed smartphone position is feasible and assessable for evaluating CPR quality and common mistakes during V-CPR. This pilot study aimed to measure the feasibility of V-CPR when a lay responder is alone with the victim in an OHCA situation. We explored three possible influencing factors in this pilot study.

1. Camera placement and perspective (the feasibility of an appropriate smartphone position).
2. Camera placement time (the time until the lay responder can place the smartphone and start chest compressions).
3. The quality and assessability of the video call and chest compression (based on assessors evaluation).

## Methods

To learn more about the feasibility of V-CPR when the lay responder was alone, we conducted a randomized controlled pilot study covering all the aforementioned factors. The Consolidated Standards of Reporting Trials (CONSORT) reporting guideline was followed^18^. The research complies with all relevant ethical regulations. The study was conducted according to the principles of the Declaration of Helsinki (the most recent version established at the 64th WMA General Assembly, Fortaleza, Brazil, October 2013) and in accordance with the Medical Research Involving Human Subjects Act (WMO).

Our study was retrospectively registered at ClinicalTrials.gov (registration number: NCT06794398, date of first registration: 01/27/2025). The detailed study protocol is presented in Supplementary Material 1.

### Ethics

The study protocol was approved by the Institutional Ethics Committee of the University of Pécs (approval number: PTE/87175-1/2022). All participants received detailed information about the research and signed a declaration of informed consent if they agreed to the research conditions and were willing to participate. Participants were informed of their right to quit at any time during the study, with no personal consequences. All participants provided written consent to publish their photographs in the manuscript. Additional written informed consent was obtained from all participants for the publication of images in which they may be identifiable, in this open-access article.

### Participants, time, and location

University workers from the University of Pécs Faculty of Health Sciences (Hungary) were involved in our pilot study as first responders. Eligible for inclusion were all types of university workers. Participants who were not capable to perform CPR (e.g. physical or mental impairment) were excluded from the study. Participants did not receive any compensation for their participation.

In addition, qualified Basic Life Support (BLS) instructors were involved in our study to evaluate the quality and assessability of the videos. Data were collected in July 2024. The pilot study was conducted at the Human Patient Simulation Center of the University of Pécs Faculty of Health Sciences.

To enhance clarity, we would like to define the different roles of participants and contributors at this point. Individuals who positioned the smartphone appropriately during the scenario and subsequently performed chest compressions are referred to as *participants*. *Dispatcher* provided instructions to the participants via live video connection from a separate room. A technical *operator* was present in the room for documentation purposes only; they took photographs of the various settings to capture the camera positions established by the participants. After the scenario—which was recorded—*assessors* reviewed the video records and assessed the recordings according to predefined criteria.

### Randomization and blinding

Each participant completed the full scenario two times in a randomised fashion (5 participants started inside and 5 started outside; and then they repeated in the other area). Simple randomization method was used by computer generated random numbers when participants arrived (even—starting inside, odd—starting outside). After randomization, study participants were informed about their task, but were blinded to the purpose of the study. The dispatchers, assessors and the operator were informed about their tasks in the different groups but were blinded to the study design and the outcomes.

### Measurement and data collection

We prepared two standardized locations: a residential living room (representing OHCA at home and inside) and a yard of a house (representing OHCA outside). The living room was selected because it typically offers a variety of fixed and movable objects that could facilitate optimal camera placement. The yard was included based on our assumption that it would present more limited options in this regard. In addition, environmental and technical factors differ between this two locations (e.g. light, internet signal, etc.) giving the opportunity for comparison. An actor was placed in both areas to represent OHCA victims. The prepared locations are listed in Supplementary Material 1 (Fig.A.1 and Fig.A.2). For the standard preparation of the actor’s position in each scenario, we marked the actor’s location using adhesive tape.

Another room was prepared for the dispatcher center. Two people supported the data collection process: an experienced BLS instructor played the role of the dispatcher in a separate room equipped with a laptop (ASUS Expert Book, ASUSTek Computer Inc., Taipei, Taiwan) and good-quality WiFi Internet access; the study operator was equipped with a smartphone (Xiaomi Redmi Note 13, Xiaomi Inc., Beijing, China), providing a live video call between the smartphone (5G Internet access) and the dispatcher’s laptop (WiFi Internet access mentioned above). Video calls were made using Microsoft Teams software (Microsoft Corporation, Redmond, WA, USA). During the scenario, there was no communication or help related to the camera placement between the operator, dispatcher, and study participants (except for providing standardized information to the participants, see below).

#### Camera placement and perspective

**Fig. 1:**
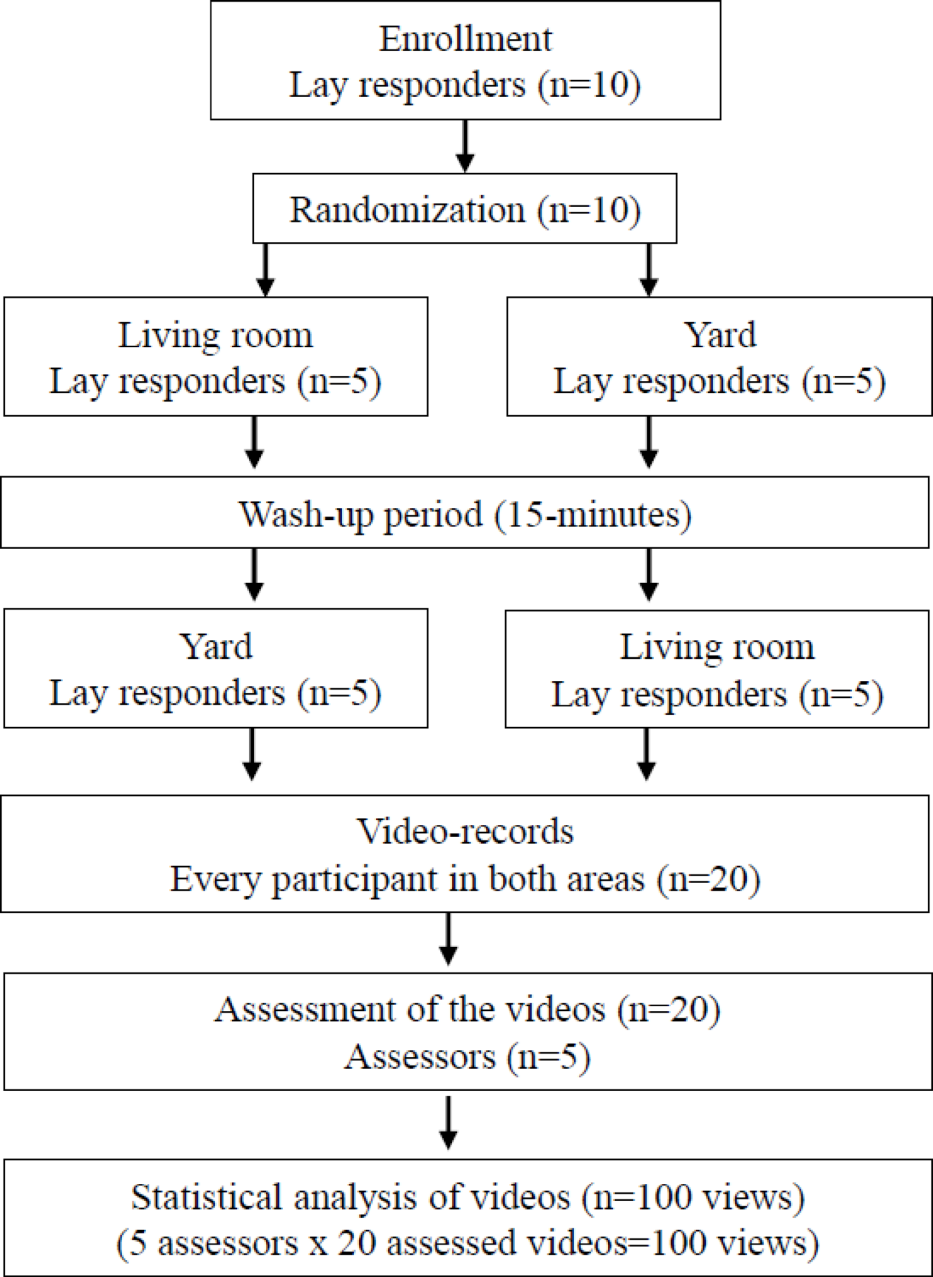
Study flow-chart.

Each participant performed the tasks in both areas after preparation. A randomized controlled crossover design was performed: five participants started in the residential living room, and five participants started in the yard (after a 15-minute wash-up period, they visited the other area) (Fig. 1). The basic situation was stated by a study operator: “Entering the venue you will find a person suffering from OHCA. The patient did not have normal breathing; therefore, starting chest compression was necessary. You will receive instructions via a live video call with this smartphone.” After entering the room, a live video connection was established (the dispatcher called the participant, who should connect with the smartphone). The dispatcher gave the following information to the participants: “You should find an appropriate smartphone position that ensures the visibility of chest compression provided by you. Any object can be used in the current environment. If you are ready, please put your hands on the victim’s chest.” After successful smartphone placement, the study operator terminated the task and photographed the camera placement and participant’s position. In addition, a screenshot of the dispatcher’s laptop screen was captured to document the dispatcher’s visual perspective during the scenario.

#### Camera placement time

The entire video call between the participant and the dispatcher was recorded using Microsoft Teams. Camera placement time was subsequently measured based on the recording. Specifically, the time interval was defined as the duration between the dispatcher’s initial instruction and the participant’s first chest compression attempt (i.e., placing their hands on the victim’s chest).

#### Quality and assessability of the video

After camera placement, the actor was changed by a Resusci Anne CPR manikin connected to the QCPR software (Laerdal, Stavanger, Denmark). To minimize bias, we collected an actor with physical parameters similar to those of the CPR manikin. In addition, participants and previously installed smartphone positions did not change. After that, the dispatcher gave some basic information: “Please place your hand on the lower half of the patient’s sternum and provide high-quality chest compressions with a depth of 5 to 6 cm, a rate of 100–120 min^− 1^.” Instructions for performing high-quality chest compressions were provided in accordance with the latest ERC guidelines^19^. In the absence of an established V-CPR protocol in Hungary the standardized T-CPR protocol was adapted for use during the scenarios^20^. Similarly to our previous study, this adaptation was supplemented with real-time visual feedback, utilizing the live video stream to offer more targeted guidance, such as corrections related to hand placement or other aspects of chest compression quality^21^. The participants were provided chest compressions for 30 s. Data were recorded using the QCPR software. After chest compressions, the study operator terminated the procedure.

After the scenarios, the participants completed a short questionnaire on their experiences during the study, related to the first outcome (Supplementary Material 1, Table A.1).

The entire video call between the participants and dispatcher was recorded. To explore the assessability of the videos (general quality of the video, visibility, chest compression quality), we prepared a short version containing only 30-s long chest compressions. To improve realism, we used the original videos and did not change, or improve their quality. In total, 20 videos were analyzed (ten in the living room and ten in the yard).

Subsequently, five assessors (experienced in CPR training) assessed the videos. All assessors watched all 20 videos, but the order in which the videos were played was randomly changed. They were able to watch all the videos twice (the first time to assess the general quality of the video, the second time to assess chest compression parameters), and then they filled out the checklist prepared by the authors (quality and assessability of the videos) (Supplementary Material 1, Table A.2).

In addition, some demographic data of the assessors were recorded: gender, age, CPR training experience years, and online practical CPR training experience (yes or no).

To assess the accuracy of the assessment, previously recorded QCPR data (related to chest compression depth and rate) were used. Related to hand position and placement, the study operator noted the correctness/incorrectness during the 30-s scenario; these data were used for evaluation.

High-quality chest compression was accepted as described in the current ERC 2021 Guidelines: interlocked fingers, hand placed on the lower half of the sternum, depth of 5–6 cm, rate of 100–120 min^− 1^^19^. Supplementary Material 1 (Table A.3) presents the quality of chest compressions in the 20 videos collected. These data were comparable to the assessors’ answers. The assessors evaluated the videos using the same laptop used by dispatchers. The assessment process of the assessors was monitored by the study operator. For the subgroup analysis, 100 video views were analyzed (5 assessors × 20 videos = 100 video views).

### Outcomes

Our pilot study revealed the following outcomes.

*Outcome 1: Camera placement*,* and perspective*.

- Camera pictures transferred via Microsoft Teams from the smartphone to the laptop were recorded (to see what the dispatcher sees).
- A picture or photo of the environment was taken (to see the smartphone’s position in the area and the used object to stabilize it).
- Experiences and opinions of the participants regarding the feasibility of V-CPR and challenges during the scenarios (based on the short questionnaire).

*Outcome 2: Camera placement time*.

- The entire video call between the participant and dispatcher was recorded using Microsoft Teams. The camera placement time was then measured. The time was detected from the dispatcher’s instructions to the first chest compression (putting the hand on the patient’s chest) based on the recorded video.

*Outcome 3: Quality and assessability of the video*.

- Measure the general quality of the video (chest compression parameters, clarity, lagging) based on assessor answers.
- Assessability based on assessor answers.
- Assessor accuracy (chest compression quality).
- Subgroup comparisons (living room vs. yard, camera placement, and position, lighting, and used object).

### Statistical analysis

Descriptive statistics were used to describe the participants. The study parameters were assessed for normal distribution and reported as numbers (percentages) and means (SDs). The study parameters were assessed for normality using the Shapiro–Wilk test. Continuous variables were compared using the t-test if a normal distribution was observed. If the distribution was not normal, Mann-Whitney-test was used. Categorical variables were compared using the chi-square test or Fisher’s exact test, as appropriate. A two-tailed p-value of < 0.05 was considered statistically significant. Statistical analyses were conducted using SPSS 26.0 (Statistics Package for Social Sciences, Chicago, IL, USA).

## Results

### Participants flow, recruitment, and baseline characteristics.

In total, 10 people (two teachers experienced in cardiopulmonary resuscitation (CPR) training, one lawyer, two staff members of a simulation center, one IT specialist, and four administrative colleagues; only the two teachers were familiar with the topic of CPR) from the University of Pécs Faculty of Health Sciences (Hungary) were involved in our pilot study as first responders (participants). None of the participants were excluded before the randomization.

In addition, five qualified Basic Life Support (BLS) instructors were involved in our study to evaluate the quality and assessability of the videos (assessors). Table 1 shows the characteristics of the study population. The study flowchart is visible in Fig. 1.

**Table 1:** Main characteristics of the study population (lay responders and assessors). CPR = cardiopulmonary resuscitation; V-CPR = video-assisted cardiopulmonary resuscitation; sd = standard deviation.

Lay responders	*N* = 10
Male, n (%)	5 (50)
Mean age, years (SD)	35.6 (10.1)
CPR training < 1 year, yes, n (%)	3 (30)
Prior information about V-CPR, yes, n (%)	3 (30)

Assessors	*N* = 5
Male, n (%)	2 (40)
Mean age, years (SD)	35.6 (3.1)
CPR teaching experience, years (SD)	10.4 (4.9)
Online practical CPR training experience, yes, n (%)	2 (40)

### Primary outcome-camera placement and perspective

**Fig. 2:**
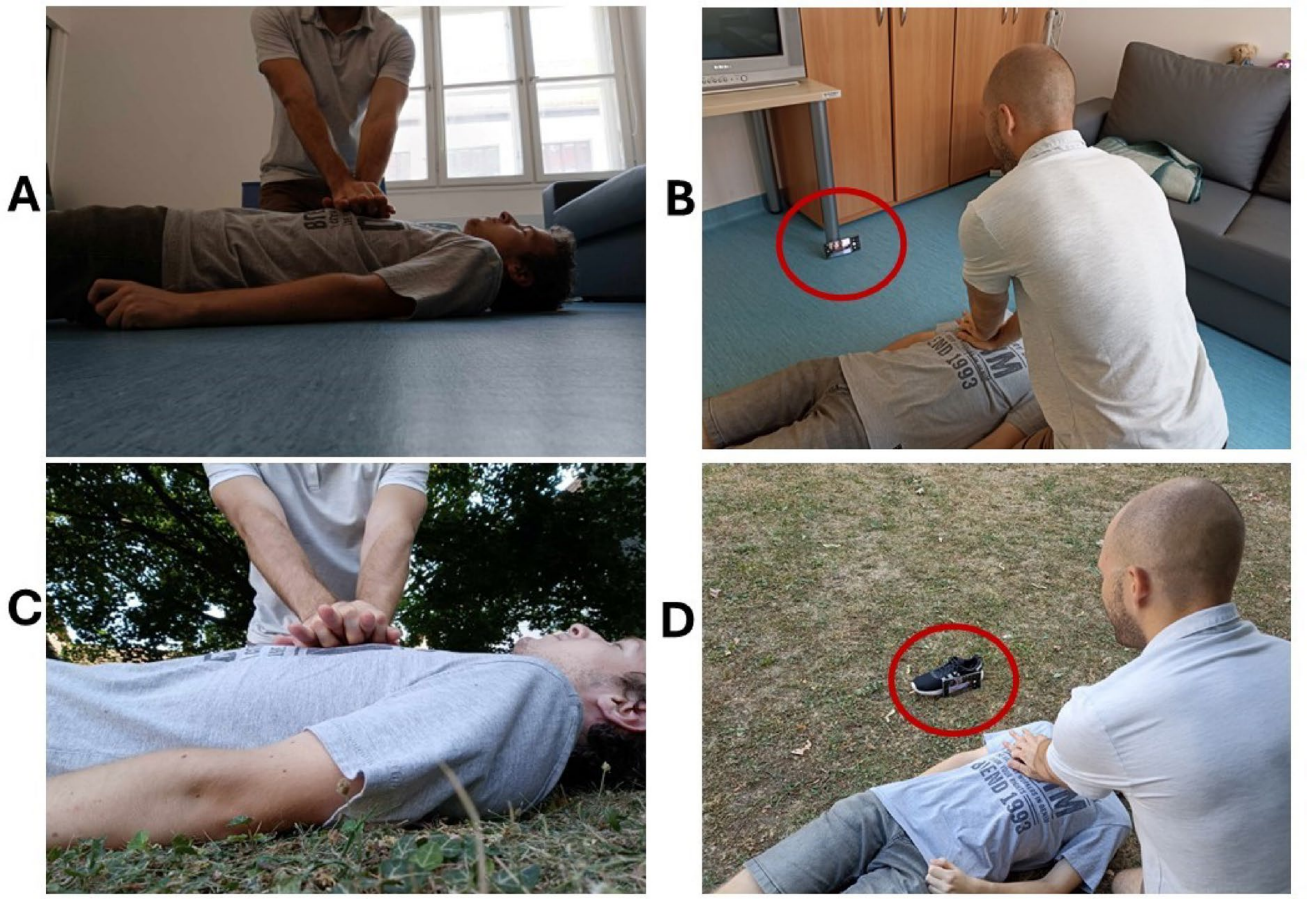
Two feasible ways to place the smartphone when the lay responder is alone with the victim. (**A**)  Smartphone’s camera picture via video call (living room, inside); (**B**)  smartphone’s placement (living room, inside); (**C**) smartphone’s camera picture via video call (yard, outside); (**D**)  smartphone’s placement (yard, outside). Red marks on B and D show the smartphone and the used object to stabilize it: a table leg in (**B**), and the victim’s shoe in (**D**).

Every participant could place the smartphone and provide a live video of chest compressions to the dispatcher in both areas. To present the results, we paired pictures of the smartphone’s front camera from an environmental perspective [Supplementary Material 2 (Fig. B.1–10)]. Participants mainly used fixed objects in the living room (e.g., a table leg) and moveable objects (e.g., a shoe) in the yard. Figure 2 shows two examples (one in the living room and one in the yard) of smartphone placement that may be feasible for real implementation In these cases the device was placed on the ground around 1 m distance from the victim, oriented vertically, with the patient shown from the side. The camera faced the responder, whose arms were visible in the frame, while the patient’s chest and entire torso, as well as parts of the head and legs, were also captured.

After the scenarios, all participants rated the V-CPR technology highly useful. The mean score of nervousness was 1.5 (SD = 0.8) (out of 4, where 1 is no nervousness, 4 is a very high level of nervousness) in the first scenario, as well as in the second scenario. Nine out of ten participants answered that the outside area was more challenging (because of the lower number of moveable objects, voice and picture impairments, and lower quality internet connection).

### Secondary outcome-camera placement time

The time was detected from the dispatcher’s instructions to the first chest compression based on the recorded video (Supplementary Material 1). Table 2 lists the times in both locations and the environmental objects used to stabilize the smartphone. The mean time of placing the camera was 23.4 s (SD = 16.6) in the living room, and 34.0 s (SD = 16.6) in the yard. No statistical difference was found between smartphone placement times related to the location (*p* = 0.075).

**Table 2:** The time required to place the smartphone and the used objects to stabilize it. Data are not normally distributed. To compare the mean times, Mann–Whitney U-test was used.

Responder no.	Location				*p*-value
	Residential living room		Yard		
	Used object to stabilize the smartphone	Time to the first chest compression (sec)	Used object to stabilize the smartphone	Time to the first chest compression (sec)	
1	Table leg	35	Backpack	23	
2	Table leg	14	Flower holder	27	
3	Pitcher, mug (elevated position on a table)	40	Shoe	25	
4	Pitcher	16	Flower holder and a shoe	60	
5	Table leg	53	Stone	40	
6	Table leg	16	Flower holder	22	
7	Chair	18	Stone	25	
8	Table leg	16	Brick	33	
9	Table leg	23	Stone	39	
10	Table leg	28	Bucket	61	
Mean time, sec (SD)		23.4 (16.6)		34.0 (16.6)	0.075

SD, standard deviation.

### Tertiery outcome-quality and assessability of the video

Table 3 summarizes the assessors’ answers for all 20 videos. The mean score of the general video quality was 2.7 (SD = 0.8) out of 4. The assessors considered chest compression depth [3.1 (SD = 0.8)] and hand placement on the chest [2.9 (SD = 0.6)] to be the least assessable, and hand position with interlocked fingers [3.6 (SD = 0.5)] and chest compression rate [3.6 (SD = 0.9)] to be the most assessable. Correctness was associated with it: the success rate of assessing hand position and chest compression rate was 86% and 84%, respectively; chest compression depth was correctly assessed in only 32% of the cases.

**Table 3:** Summary of the assessability and quality of the videos.

Video No.	Assessability of the chest compression				Correctness of the assessment				Video quality			
	Hand position, mean (SD)	Hand placement, mean (SD)	CC depth, mean (SD)	CC rate, mean (SD)	Hand position, *n* (%)	Hand placement, *n* (%)	CC depth, *n* (%)	CC rate, *n* (%)	Clarity of the picture, mean (SD)	Lagging, mean (SD)	Camera perspective, mean (SD)	General quality, mean (SD)
1	2.2 (1.1)	2.2 (0.4)	2.8 (0.4)	3.2 (0.4)	3 (60)	5 (100)	2 (40)	4 (80)	1.8 (0.8)	2.6 (0.5)	2.6 (0.5)	2.0 (0.7)
2	3.8 (0.4)	3.2 (1.1)	3.8 (0.4)	4.0 (0.0)	5 (100)	4 (80)	4 (80)	4 (80)	3.8 (0.4)	3.6 (0.5)	3.2 (1.1)	3.4 (0.5)
3	3.4 (0.8)	2.2 (0.8)	1.6 (0.9)	1.2 (0.4)	5 (100)	0 (0)	2 (40)	5 (100)	1.4 (0.5)	1.0 (0.0)	1.8 (0.8)	1.2 (0.4)
4	4.0 (0.0)	3.2 (1.1)	3.8 (0.4)	4.0 (0.0)	5 (100)	4 (80)	0 (0)	4 (80)	3.4 (0.9)	3.4 (0.5)	3.4 (0.9)	3.4 (0.5)
5	4.0 (0.0)	3.4 (0.5)	3.6 (0.5)	4.0 (0.0)	5 (100)	5 (100)	0 (0)	5 (100)	2.4 (0.9)	3.2 (1.1)	3.2 (0.4)	2.8 (0.8)
6	3.4 (0.5)	2.6 (0.5)	2.8 (0.4)	3.4 (0.5)	4 (80)	5 (100)	2 (40)	5 (100)	2.4 (0.9)	2.8 (0.8)	2.4 (0.5)	2.2 (0.8)
7	4.0 (0.0)	3.6 (0.5)	3.8 (0.5)	4.0 (0.0)	5 (100)	5 (100)	3 (60)	4 (80)	3.8 (0.5)	3.6 (0.5)	3.6 (0.5)	3.4 (0.5)
8	3.8 (0.5)	3.8 (0.5)	3.2 (0.8)	4.0 (0.0)	4 (80)	5 (100)	1 (20)	4 (80)	3.4 (0.5)	3.8 (0.5)	3.6 (0.5)	3.6 (0.5)
9	3.6 (0.5)	3.0 (1.0)	3.6 (0.9)	4.0 (0.0)	5 (100)	3 (60)	1 (20)	3 (60)	2.8 (0.5)	2.4 (0.9)	3.0 (0.7)	2.4 (0.5)
10	2.8 (1.3)	2.2 (1.3)	2.2 (1.1)	3.8 (0.5)	2 (40)	3 (60)	1 (20)	4 (80)	2.8 (0.8)	3.0 (0.0)	2.2 (1.3)	2.6 (1.1)
11	4.0 (0.0)	3.2 (1.1)	3.8 (0.4)	4.0 (0.0)	5 (100)	4 (80)	0 (0)	4 (80)	3.4 (0.9)	3.4 (0.5)	3.4 (0.9)	3.4 (0.5)
12	3.8 (0.4)	3.2 (1.1)	3.8 (0.4)	4.0 (0.0)	5 (100)	4 (80)	4 (80)	4 (80)	3.8 (0.4)	3.6 (0.5)	3.2 (1.1)	3.4 (0.5)
13	3.6 (0.5)	3.0 (1.0)	3.6 (0.9)	4.0 (0.0)	5 (100)	3 (60)	1 (20)	3 (60)	2.8 (0.5)	2.4 (0.9)	3.0 (0.7)	2.4 (0.5)
14	3.8 (0.5)	3.8 (0.5)	3.2 (0.8)	4.0 (0.0)	4 (80)	5 (100)	1 (20)	4 (80)	3.4 (0.5)	3.8 (0.5)	3.6 (0.5)	3.6 (0.5)
15	3.4 (0.5)	2.6 (0.5)	2.8 (0.4)	3.4 (0.5)	4 (80)	5 (100)	2 (40)	5 (100)	2.4 (0.9)	2.8 (0.8)	2.4 (0.5)	2.2 (0.8)
16	2.2 (1.1)	2.2 (0.4)	2.8 (0.4)	3.2 (0.4)	3 (60)	5 (100)	2 (40)	4 (80)	1.8 (0.8)	2.6 (0.5)	2.6 (0.5)	2.0 (0.7)
17	3.4 (0.8)	2.2 (0.8)	1.6 (0.9)	1.2 (0.4)	5 (100)	0 (0)	2 (40)	5 (100)	1.4 (0.5)	1.0 (0.0)	1.8 (0.8)	1.2 (0.4)
18	2.8 (1.3)	2.2 (1.3)	2.2 (1.1)	3.8 (0.5)	2 (40)	3 (60)	1 (20)	4 (80)	2.8 (0.8)	3.0 (0.0)	2.2 (1.3)	2.6 (1.1)
19	4.0 (0.0)	3.4 (0.5)	3.6 (0.5)	4.0 (0.0)	5 (100)	5 (100)	0 (0)	5 (100)	2.4 (0.9)	3.2 (1.1)	3.2 (0.4)	2.8 (0.8)
20	4.0 (0.0)	3.6 (0.5)	3.8 (0.5)	4.0 (0.0)	5 (100)	5 (100)	3 (60)	4 (80)	3.8 (0.5)	3.6 (0.5)	3.6 (0.5)	3.4 (0.5)
Overall	3.6 (0.5)	2.9 (0.6)	3.1 (0.8)	3.6 (0.9)	4.3 (86)	3.9 (78)	1.6 (32)	4.2 (84)	2.8 (0.8)	2.9 (0.8)	2.9 (0.6)	2.7 (0.8)

Video quality scores were given using a 4-point likert scale: 1—very bad, 2—bad, 3—good, 4—very good. The assessability of the chest compressions was evaluated using a 4-point likert scale: 1—very bad, 2—bad, 3—good, 4—very good. The correctness of the evaluation was calculated based on the prior evaluation of the authors and the QCPR software. CC = chest compression; sd = standard deviation.

Supplementary Material 3 shows the subgroup analysis. Overall, video quality and assessability had better scores in the living room than in the yard in every aspect (*p* < 0.05). However, the accuracy of the assessment did not differ between the two locations, except for hand placement (*p* = 0.001). Furthermore, differences were not identified when comparing scores based on the other subgroups (*p* > 0.05 in every case), except in one case where assessing hand placement was better with moveable objects (*p* = 0.019). In response, one of the assessors suggested that the smartphone’s ground position would be better in situations where there are more lay responders (e.g., there is no camera movement to decrease the quality, or the second responder can wait for the ambulance or bring the defibrillator).

## Discussion

In this article, we studied possible methods for the feasibility and assessability of V-CPR when the lay responder is alone with an OHCA patient. We highlighted three factors: (1) camera placement and perspective, (2) camera placement time (time from the dispatcher’s instructions to the first chest compression), and (3) quality and assessability of the video picture. To the best of our knowledge, this is the first study to investigate V-CPR technology in lay responders alone.

V-CPR is a relatively recent approach that enables dispatchers to visually assess the scene and the victim, allowing them to provide more accurate instructions and feedback to lay responders compared to traditional T-CPR^9–12^. Recent studies have demonstrated improved clinical outcomes and more effective chest compressions when using V-CPR compared to unassisted CPR or T-CPR^13–15^.

A significant proportion of OHCAs occur in residential locations^22^. Cardiac arrest in residential locations has a much lower survival rate than cardiac arrest in public locations^23,24^. If these OHCAs are witnessed, a second person may not always be present, thereby reducing the usability of certain technologies (e.g., V-CPR). For this reason, we chose residential locations (living rooms and yards) for our pilot study.

Several technical factors may influence the feasibility and effectiveness of V-CPR, including camera positioning, video quality, poor signal, and environmental conditions such as excessive brightness, darkness, or inclement weather^10,16,17^. Furthermore, in real-life situations, V-CPR is typically feasible only when at least two lay responders are present—one to perform CPR and the other to hold the camera. Only a few studies have examined the technical aspects of V-CPR^16,17,25^. Most prior simulation studies either used a pre-positioned tripod or involved a study team member to achieve the desired camera angle; in real scenarios, the second lay responder typically held the device^10^. The best camera placement was on the opposite side of the CPR provider, 140 cm above ground^16^. However, a second person may not always be available in actual emergencies. In our study, where each lay responder was alone with the victim, every participant was able to find a solution for placing the camera in both simulated settings. In the living room, participants primarily relied on fixed objects (e.g., a table leg), while in the yard they mainly used movable items (e.g., a shoe) to achieve a suitable camera angle. Although it is possible to customize camera positioning even when only one responder is present—especially if movable objects are available—such positioning is far more flexible when two or more responders are involved, as one of them can adjust the device dynamically during the call. Only one of the 10 participants used a similarly elevated position (around 40 cm high), whereas the others placed the smartphone on the ground. No differences were observed between the two placement methods. In these latter cases, the typical camera position was as follows: the device was placed on the ground around 1 m distance from the victim, oriented vertically, with the patient shown from the side. The camera faced the responder, whose arms were visible in the frame, while the patient’s chest and entire torso, as well as parts of the head and legs, were also captured.

In OHCA, the time to first chest compression is extremely important. This time depends on many factors: time from collapse to recognition of cardiac arrest, time until calling the local emergency number, time of communication with the dispatcher, primary assessment of the patient, and starting chest compression^26^. Dispatcher-assisted CPR methods (T-CPR and V-CPR) were associated with higher survival rates^10,27^. In our scenario, participants were informed from the outset that the victim was unresponsive and not breathing (i.e., they did not need to assess responsiveness or breathing). The scenario began with an established video connection, during which participants were instructed to position the camera so that the dispatcher could clearly observe the chest compressions. It took approximately 30 s on average. This delay could have been avoided if two responders were present, as one could have started compressions while the other positioned the camera. Previous research has similarly shown that time to first chest compression can be longer when using V-CPR or T-CPR; however, the ability to follow instructions and receive corrective feedback may compensate for this initial delay during the course of the resuscitation attempt^21,28,29^. As comprehensive measurement of time-related variables was not the aim of this study, and given the limited available data and methodological differences across studies, comparability with previous research is inherently challenging.

Following the scenarios, all participants rated the V-CPR technology as highly useful. The average self-reported nervousness score was 1.5 out-of 4 in both scenarios. Nine out of ten participants reported that the outdoor setting was more challenging, citing reasons such as the limited availability of movable objects, impaired audio and video quality, and weaker internet connectivity.

The quality and assessability of video calls are essential for V-CPR. A previous study did not find differences between low-, mid-, and high-resolution videos related to assessability^17^. However, using V-CPR does not automatically improve CPR, and dispatchers should be trained^30^. In our pilot study, five qualified BLS instructors (assessors) assessed the videos recorded during the scenarios. According to feedback from the assessors, the yard environment was rated as less assessable due to several factors: video interruptions caused by weaker internet connectivity, lower image quality, and variable lighting conditions (e.g., excessive brightness, shadows) that impaired visibility. Additionally, the larger open space made verbal communication more difficult due to reduced audibility. Despite this subjective difference, the number of correctly or incorrectly identified chest compression parameters was similar at both locations. The assessors found chest compression depth and hand placement on the chest to be the most difficult elements to evaluate, while hand position with interlocked fingers and chest compression rate were judged to be the most assessable. This was reflected in the accuracy of their evaluations: hand position and compression rate were correctly assessed in 86% and 84% of cases, respectively, whereas chest compression depth was accurately evaluated in only 32% of cases. These rates were similar to those in previous studies; however, previous studies examined correctness with at least two lay responders and not fixed camera placement by the sole lay responder^13,25^. The limited accuracy in assessing chest compression quality may be attributed to factors such as video quality, the characteristics of the camera position established by the participants (e.g., distance, angle), or the assessors’ limited experience with online evaluation. These issues could potentially be improved through targeted dispatcher training and the implementation of standardized protocols for optimal camera positioning. Additionally, integrating artificial intelligence technologies into the assessment process may also prove beneficial.

Our study has several limitations. Our sample size was very small and the sample did not represent the general population (lay responders or assessors). Since our study had a technical focus, we did not measure the entire OHCA situation and reactions of lay responders (only camera placement and chest compressions). Interestingly, study participants answered they were not really nervous, this does not seem to transfer to the real-life scenario. We prepared two general areas; however, real-life locations can vary significantly. We used certain types of devices (smartphones, laptops) and Internet connections; however, the video quality could differ when using other types of devices and/or in case of different network strenght. Our study did not contain the quality and assessability of two-person V-CPR as a control group.

## Conclusions

Our pilot study suggests that V-CPR may be feasible even when the lay responder is alone. The participants were able to acceptably place the camera using different environmental objects to stabilize their smartphones. However, camera positioning and initiating chest compressions required additional time when only a single responder was present. The quality of the videos varies between locations. Chest compression rate and hand position could be evaluated well. Correct assessment of chest compression depth was the most challenging. These results are promising; however, further randomized controlled studies should investigate how the method we examined compares to existing V-CPR approaches involving two responders. Further exploration of single-rescuer scenarios is also warranted, focusing on aspects such as: which camera position is more effective—ground-level or elevated; how the time required for camera placement can be minimized to allow earlier initiation of chest compressions; how different camera distances and angles affect video assessability to determine optimal positioning; and to what extent targeted dispatcher training can enhance the overall effectiveness of V-CPR.

## Electronic supplementary material

Below is the link to the electronic supplementary material.

Supplementary Material 1.

## Data Availability

The datasets used and/or analysed during the current study are available from the corresponding author on reasonable request.
