# Supplementary material for: Feasibility of video-assisted cardiopulmonary resuscitation when the lay responder is alone with the victim: a randomized controlled crossover pilot study
Source: Sci Rep. 2025 Jul 19;15:26268. doi: 10.1038/s41598-025-12014-6 (PMC12276233; doi:10.1038/s41598-025-12014-6)
Supplement: Supplementary file 1 — Supplementary Material 1. [file 41598_2025_12014_MOESM1_ESM.docx]

**Title:** Feasibility of video-assisted cardiopulmonary resuscitation when the lay responder is alone with the victim – a randomized controlled crossover pilot study.

**Supplementary material 1: Pilot study protocol**

***Aim of the pilot study***

This pilot study aims to create a framework of opportunities about the feasibility of video-assisted cardiopulmonary resuscitation (V-CPR) when the lay responder is alone with the victim in an out-of-hospital cardiac arrest (OHCA) situation.

We measured three possible influencing factors:

1. Measure the feasibility of appropriate smartphone position when the lay responder is alone with the victim in an OHCA situation
2. Measure the time until the lay responder can place the smartphone when he/she is alone with the victim in an OHCA situation
3. Evaluate the quality and assessability of the video call between the lay responder and the dispatcher when the smartphone’s placement was made by the lay responder who is alone with the victim in an OHCA situation.

***Ethics***

The study protocol was approved by the Institutional Ethics Committee of the University of Pécs (approval number: PTE/87175-1/2022). All participants received detailed information about the research and signed the declaration of informed consent if they agreed to the research conditions and were willing to participate. Participants were informed of their right to quit at any point during the study with no personal consequences. All participants gave written consent to publish their photos as part of the manuscript.

***Participants, time and location***

In total, 10 people (two teachers experienced in cardiopulmonary resuscitation (CPR) training, one lawyer, two staff members of a simulation center, one IT specialist, and four administrative colleagues) from the University of Pécs Faculty of Health Sciences (Hungary) were involved in our pilot study. Data collection was made in July 2024.

Five qualified Basic Life Support (BLS) instructors were involved in our study to evaluate the quality and assessability of the videos.

***Measurement and data collection***

The pilot study was performed in the Human Patient Simulation Center at the University of Pécs Faculty of Health Sciences. We prepared two standardized locations: a residential living room (representing OHCA at home, inside and a yard of a house (representing OHCA outside). An actor was placed in both areas representing the OHCA victim. Prepared locations are visible in Fig.A.1 and Fig.A.2.


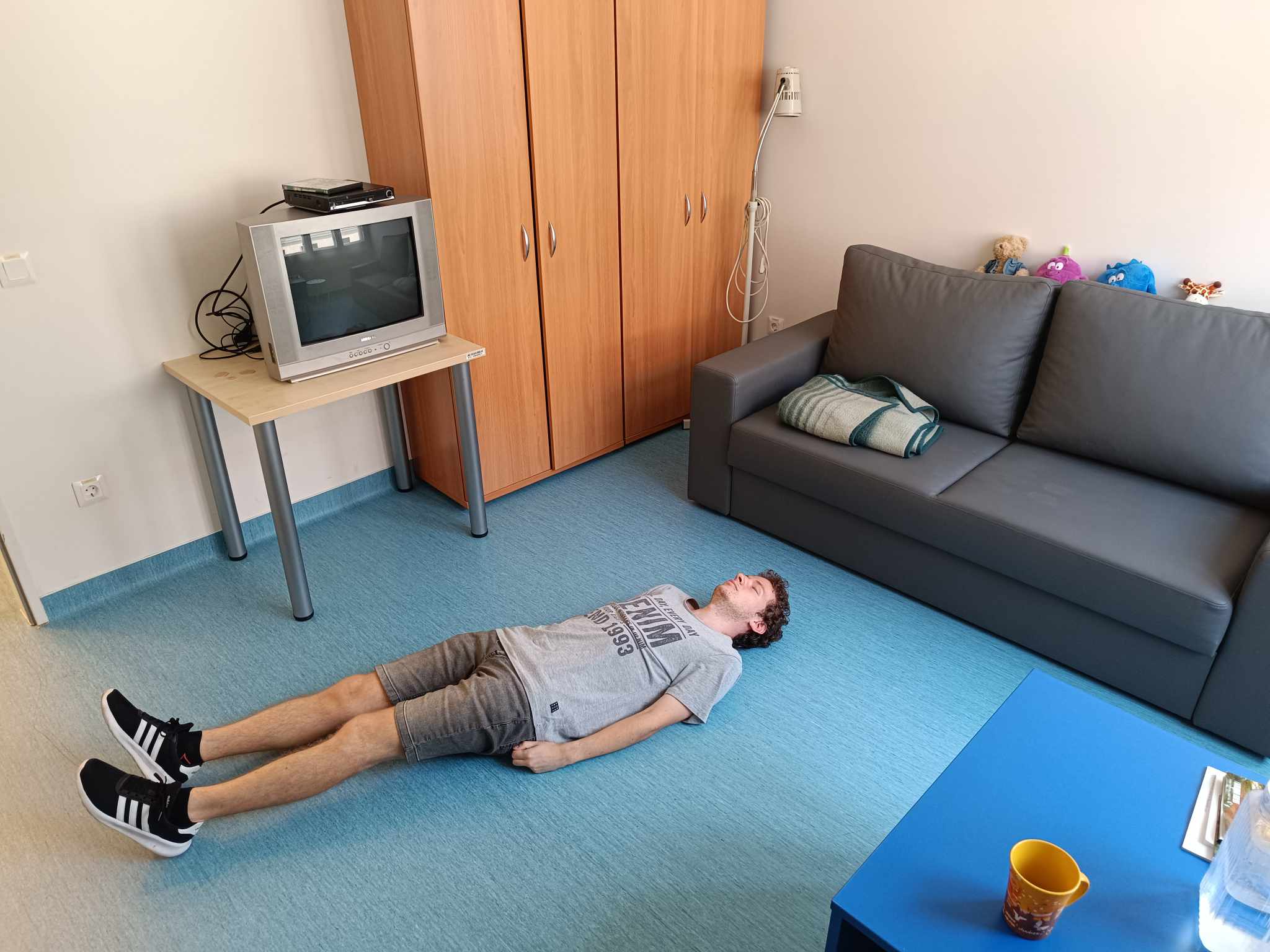


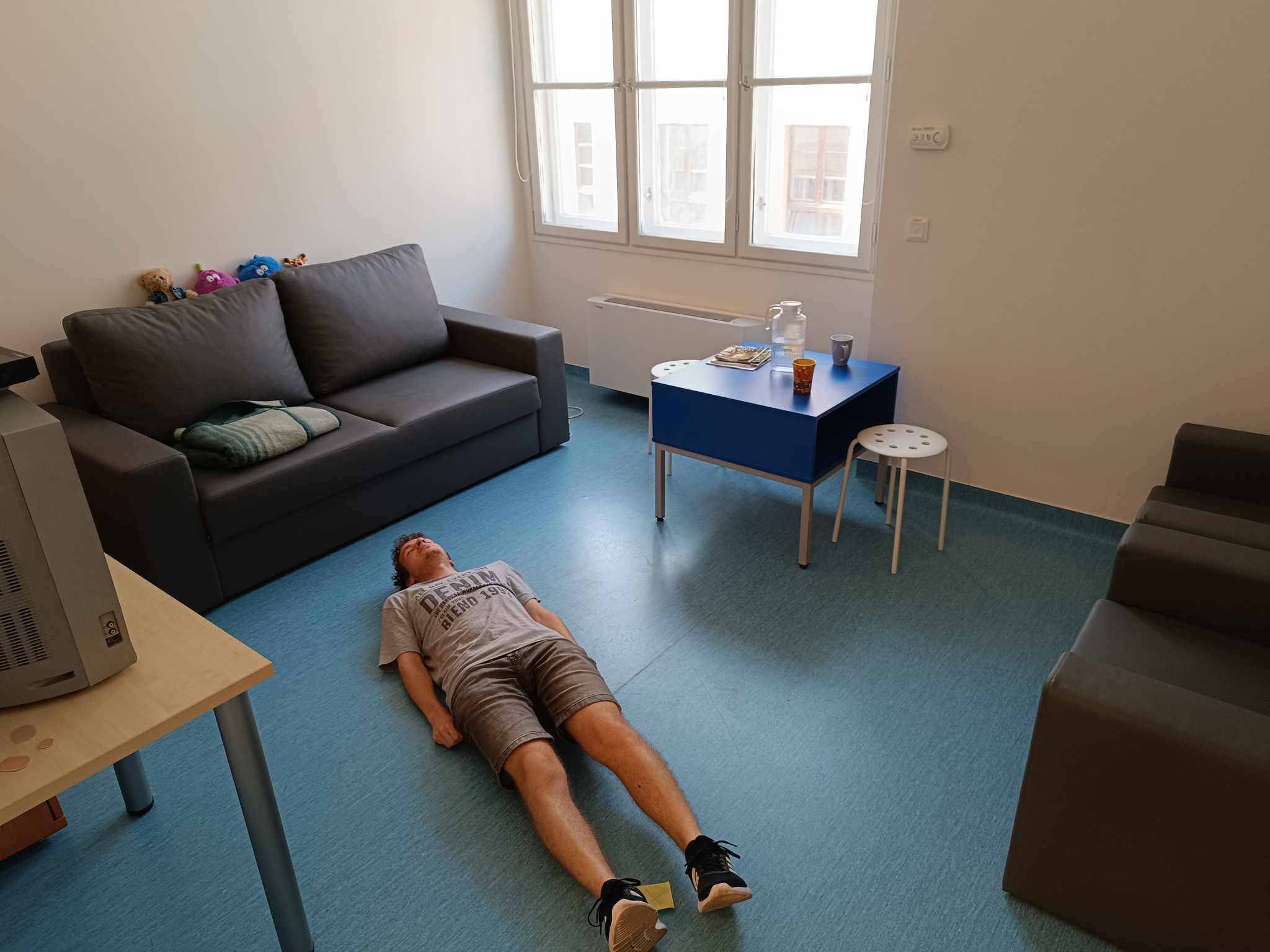


Fig.A.1. The prepared residential living room for OHCA inside situation.


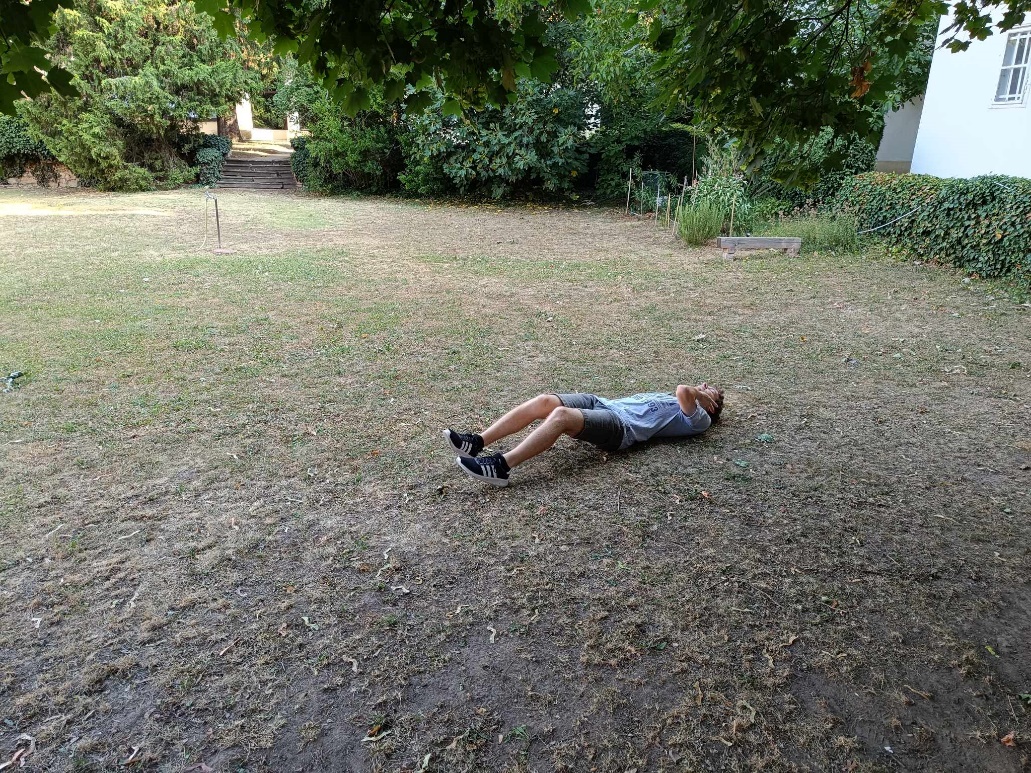

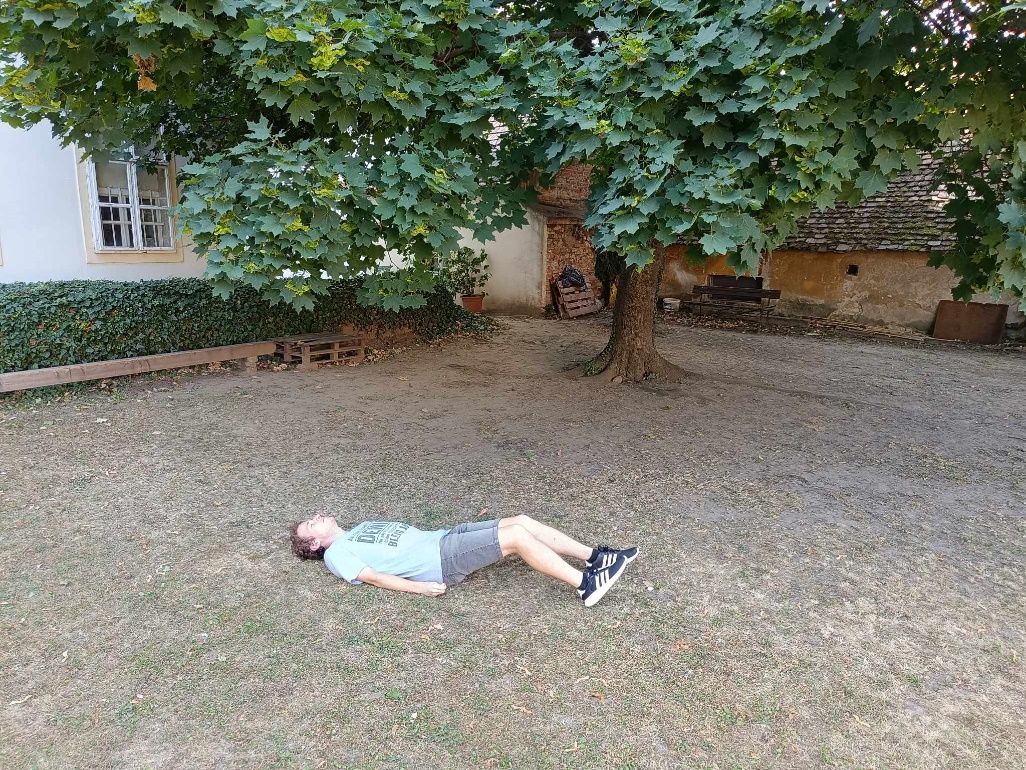


Fig.A.2. The prepared yard for OHCA outside situation.

For standard preparation of the actor’s position in each scenario, we marked the actor's place with adhesive tapes.

Another room was prepared for the dispatcher center. Two study operators supported the data collection process: an experienced BLS instructor played the role of the dispatcher in a separate room equipped with a laptop (ASUS Expert Book, ASUSTek Computer Inc., Taipei, Taiwan) and good quality Wifi internet access; the second study operator was equipped with a smartphone (Xiaomi Redmi Note 13, Xiaomi Inc., Bejing, China) providing the live video call between the smartphone (5G internet access) and the dispatcher’s laptop (Wifi internet access mentioned above). The video call was made via Microsoft Teams software (Microsoft Corporation, Redmond, USA). During the scenario, there was no communication or help between the operator and the dispatcher, and the study participants (except for giving standardized information to the participants, see below).

After preparation, each participant did the tasks in both areas. A cross-over design was performed: five participants started in the residential living room and five participants started in the yard (and after a 15-minute wash-up period they visited the other area). The basic situation was stated by a study operator: “Entering the venue you will find a person suffering OHCA. The patient has no normal breathing so starting chest compression is necessary. You will get some instructions via a live video call with this smartphone.” After entering the room, a live video connection was established (the dispatcher called the participant who should connect with the smartphone). The dispatcher gave the following information to the participants: “You should find an appropriate smartphone position that ensures the visibility of chest compression provided by you. You can use any objects in your current environment. If you are ready, please put your hands on the victim’s chest.” After successful smartphone placement, the study operator terminated the task and took a photo of the camera placement and the participant’s position.

After the camera placement, the actor was changed by a Resusci Anne CPR manikin connected to the QCPR software (Laerdal, Stavanger, Denmark). To minimize biases, we collected an actor with similar physical parameters as the CPR manikin. Meanwhile, the participant’s and the previously installed smartphone position was not changed. After that, the dispatcher gave some basic information: “Please place your hand on the lower half of the patient’s sternum and provide high-quality chest compressions with a depth of 5 to 6 cm, a rate of 100-120 min^-1^.” Participants were provided chest compressions for 30 seconds. Data were recorded by the QCPR software. After the chest compressions, the study operator terminated the process.

After the scenarios, participants filled out a short questionnaire about their experiences during our study (Table A.1).

| **Question** | **Possible answers** |
| --- | --- |
| What is your gender? | male |
|  | female |
| What is your age? | -------- years |
| Did you attend CPR training within the last year? | Yes |
|  | No |
| Did you have any information about V-CPR before this study? | Yes |
|  | No |
| How nervous were you before the first situation? | 1 – not at all nervous |
|  | 2 – low level of nervousness |
|  | 3 – middle level of nervousness |
|  | 4 – very nervous |
| How nervous were you before the second situation? | 1 – not at all nervous |
|  | 2 – low level of nervousness |
|  | 3 – middle level of nervousness |
|  | 4 – very nervous |
| How useful do you find V-CPR generally? | 1 – not at all useful |
|  | 2 – slightly useful |
|  | 3 – moderately useful |
|  | 4 – very useful |
| Which area was more challenging for you (living room vs. yard)? | Living room |
|  | Yard |
| Why? | (open answer) |

Table A.1. Questionnaire for the participants after the scenarios.

The whole video call between the participants and the dispatcher was recorded. To explore the assessability of the videos (general quality of the video, visibility, chest compression quality), we have prepared a short version containing only the 30-second-long chest compressions. To improve realism, we used the original videos and we did not change or improve the quality of these. In total, 20 videos were analysed (ten in the living room and ten in the yard).

After that, five experts (experienced in CPR training) assessed the videos. All experts watched all 20 videos but the order of playing the videos was randomly changed. They could watch all the videos two times (the first time to assess the general quality of the video, the second time to assess chest compression parameters) and then they should fill out the checklist prepared by the authors (quality and assessability of the videos) (Table A.2)

| **Task** |  | **Possible answers** |
| --- | --- | --- |
| Number of the video |  | ……… |
| Assess the evaluability and quality of the video | Hand position (interlocked fingers during chest compression) | 1 – very bad assessability |
|  |  | 2 –bad assessability |
|  |  | 3 – good assessability |
|  |  | 4 – very good assessability |
|  | Hand position (hand on the lower half of the sternum) | 1 – very bad assessability |
|  |  | 2 –bad assessability |
|  |  | 3 – good assessability |
|  |  | 4 – very good assessability |
|  | Chest compression depth | 1 – very bad assessability |
|  |  | 2 –bad assessability |
|  |  | 3 – good assessability |
|  |  | 4 – very good assessability |
|  | Chest compression rate | 1 – very bad assessability |
|  |  | 2 –bad assessability |
|  |  | 3 – good assessability |
|  |  | 4 – very good assessability |
| Assess the overall quality of the video | Image clarity | 1 – very bad quality |
|  |  | 2 –bad quality |
|  |  | 3 – good quality |
|  |  | 4 – very good quality |
|  | Video lagging | 1 – very bad quality |
|  |  | 2 –bad quality |
|  |  | 3 – good quality |
|  |  | 4 – very good quality |
|  | Smartphone position | 1 – very bad quality |
|  |  | 2 –bad quality |
|  |  | 3 – good quality |
|  |  | 4 – very good quality |
|  | Overall quality | 1 – very bad quality |
|  |  | 2 –bad quality |
|  |  | 3 – good quality |
|  |  | 4 – very good quality |
| Assess chest compression quality | Hand position (interlocked fingers during chest compression) | incorrect |
|  |  | correct |
|  |  | not assessable |
|  | Hand position (hand on the lower half of the sternum) | incorrect |
|  |  | correct |
|  |  | not assessable |
|  | Chest compression depth | too superficial |
|  |  | correct |
|  |  | too deep |
|  |  | not assessable |
|  | Chest compression rate | too slow |
|  |  | correct |
|  |  | too fast |
|  |  | not assessable |

Table A.2. Checklist for the assessors.

In addition, some demographic data of the assessors were recorded: gender, age, CPR training experience years, and online practical CPR training experience (yes or no).

To check the correctness of the assessors’ evaluation, previously recorded QCPR data (related to chest compression depth and rate) were used. Related to hand position and placement, the study operator noted the correctness/incorrectness during the 30-second scenario; these data were used for evaluation.

High-quality chest compression was accepted as described in current ERC 2021 Guidelines: interlocked fingers, hand placed on the lower half of the sternum, depth of 5-6 cm, rate of 100-120 min^-1^. Table A.3 shows the quality of chest compressions in the 20 videos. These data were comparable with the assessors’ answers. Assessors evaluated the videos using the same laptop as the dispatcher. The assessors’ evaluation process was monitored by a study operator. For subgroup analysis, 100 video views were analyzed (5 assessors x 20 videos = 100 video views).

| **Number of the video** | **Correctness of the chest compressions (CC)** | | | |
| --- | --- | --- | --- | --- |
|  | **Hand position (interlocked fingers)** | **Hand position (position on the chest)** | **CC depth** | **CC rate** |
| V1 | Correct | Not assessable | Too deep | Too slow |
| V2 | Correct | Correct | Correct | Correct |
| V3 | Incorrect | Not assessable | Too deep | Too slow |
| V4 | Incorrect | Correct | Too deep | Correct |
| V5 | Correct | Correct | Too deep | Correct |
| V6 | Incorrect | Incorrect | Too superficial | Too slow |
| V7 | Correct | Correct | Correct | Correct |
| V8 | Correct | Correct | Too deep | Correct |
| V9 | Correct | Correct | Correct | Correct |
| V10 | Incorrect | Incorrect | Too deep | Too slow |
| V11 | Correct | Correct | Correct | Correct |
| V12 | Incorrect | Not assessable | Too deep | Too slow |
| V13 | Correct | Correct | Correct | Correct |
| V14 | Correct | Correct | Correct | Correct |
| V15 | Incorrect | Correct | Too deep | Too slow |
| V16 | Incorrect | Incorrect | Too deep | Too slow |
| V17 | Correct | Correct | Too deep | Correct |
| V18 | Incorrect | Not assessable | Too deep | Too slow |
| V19 | Correct | Correct | Too deep | Correct |
| V20 | Incorrect | Not assessable | Too deep | Too slow |

Table A.3. Quality of chest compressions in the videos. Parameters were collected by the study operator (hand position and hand placement), and the QCPR software (chest compression depth and rate).

Fig.A.3 shows the study flow chart.


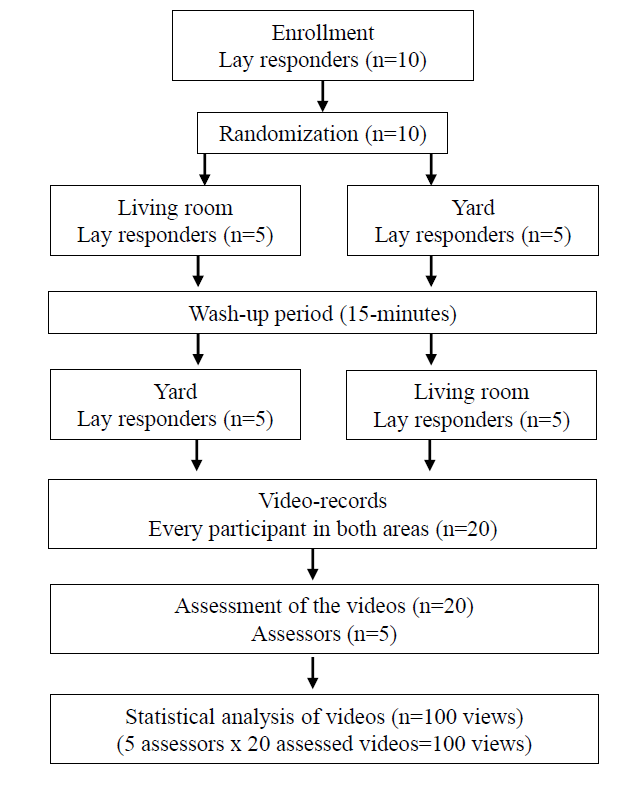


Fig.A.3. Study flow chart.

***Outcomes***

Our pilot study had three outcomes:

*Outcome 1: Camera placement and perspective*

- Camera pictures transferred via Microsoft Teams from the smartphone to the laptop were recorded (to see what the dispatcher sees)
- A picture/photo of the environment was taken (to see the smartphone’s position in the area and the used object to stabilize it)
- Experiences and opinions of the participants about V-CPR feasibility and challenges during the scenarios (based on the short questionnaire)

*Outcome 2: Time of placing the camera*

- The whole video call between the participant and the dispatcher was recorded by Microsoft Teams. The time of the camera placement was measured. Time was detected from the dispatcher's instructions to the first chest compression (putting the hand on the patient’s chest) based on the recorded video.

*Outcome 3: Assessability of the video*

- Measure the general quality of the video (chest compression parameters, clarity, lagging)
- Assessability based on the assessor's answers
- Correctness of the assessment by the assessors (chest compression quality)
- Subgroup comparisons (living room vs. yard, camera placement, lighting).

***Statistical analysis***

To describe the sample, descriptive statistics were used. Study parameters were assessed for normal distribution and reported as numbers (percentages) and means (SDs). Study parameters were assessed for normality by using Shapiro–Wilk test. If normal distribution was indicated, continuous variables were compared using t-test. If distribution was not normal, Mann-Whitney-test was used. Categorical variables were compared using Chi-square test or Fisher’s-exact test as appropriate. A two-tailed p-value of <0.05 was considered to be statistically significant. Statistical analysis was conducted using SPSS 26.0 (Statistics Package for Social Sciences, Chicago, IL, USA).

**Supplementary Material 2: Different smartphone placements by the participants**

***Camera placement***

Fig. B.1-10 show all the smartphone placements of the participants. We created a figure from four pictures of each participant:

- one picture of camera placement in the living room
- one picture from the smartphone’s camera in the living room
- one picture of camera placement in the yard
- one picture from the smartphone’s camera in the yard


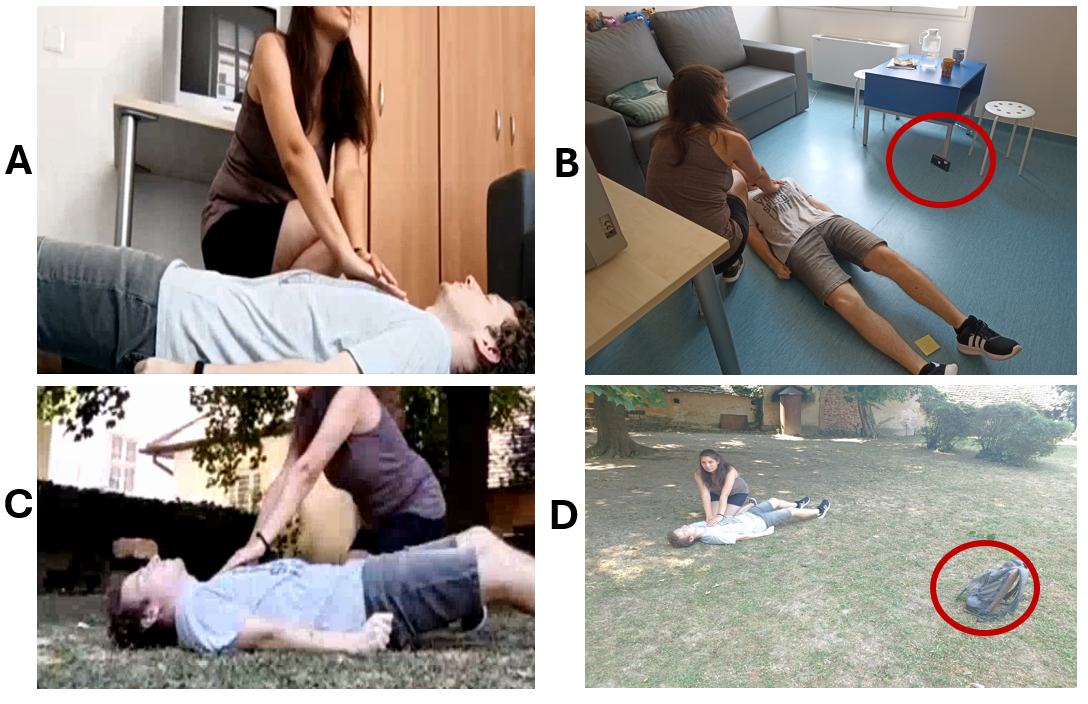
**Fig. B.1.** Results of participant No.1. A=smartphone’s camera picture via video call (in the living room); B=smartphone’s placement (in the living room); C= smartphone’s camera picture via video call (in the yard); D= smartphone’s placement (in the yard). Red marks on B and D show the smartphone and the used object (a table leg in the living room, and a backpack in the yard).


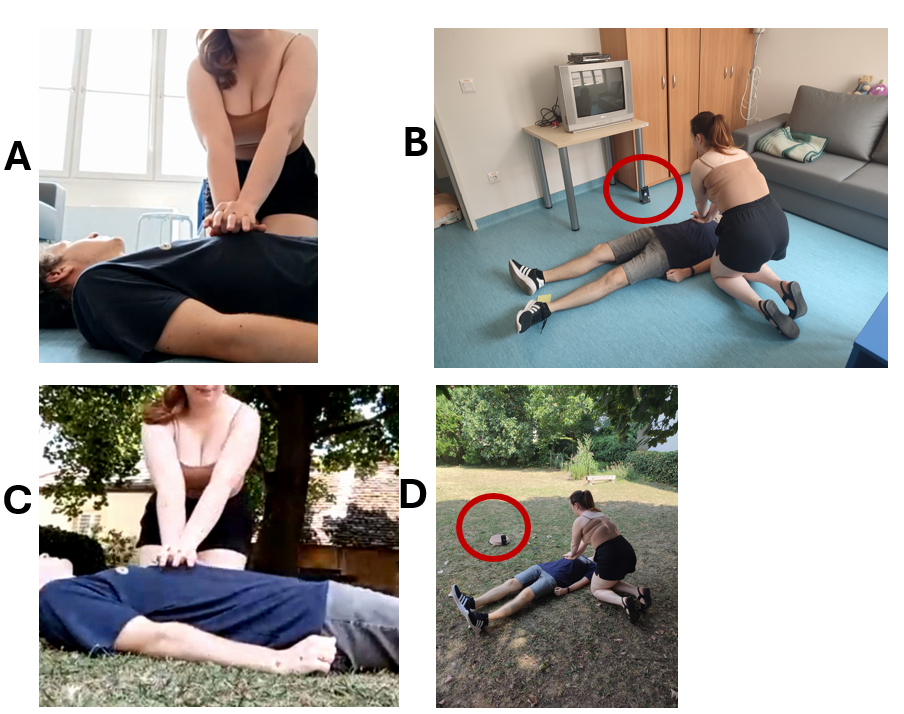
 **Fig. B.2.** Results of participant No.2. A=smartphone’s camera picture via video call (in the living room); B=smartphone’s placement (in the living room); C= smartphone’s camera picture via video call (in the yard); D= smartphone’s placement (in the yard). Red marks on B and D show the smartphone and the used object (a table leg in the living room, and a flower holder in the yard).


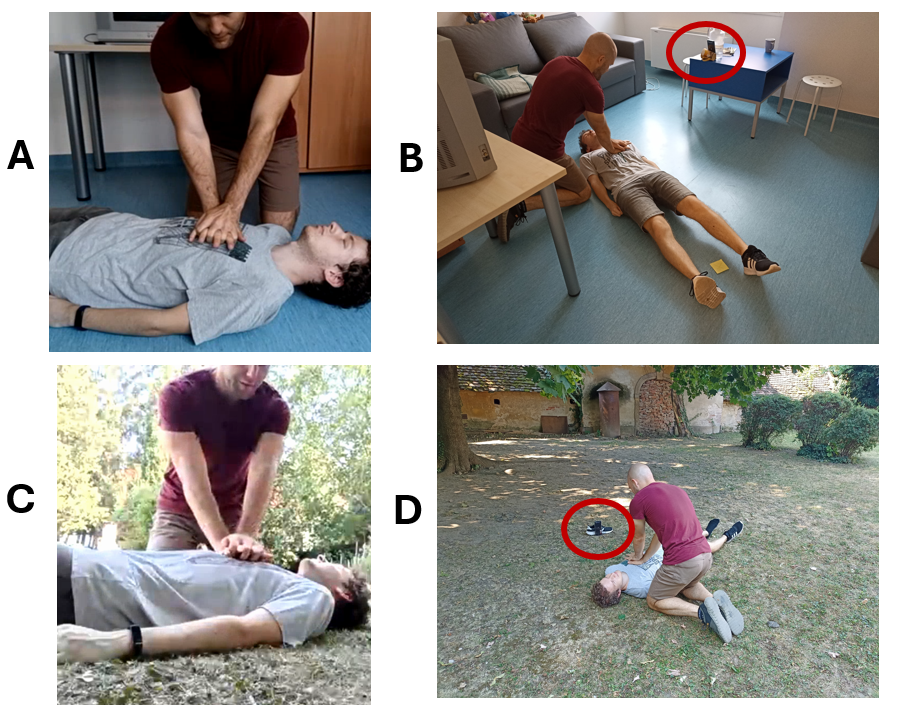


**Fig. B.3.** Results of participant No.3. A=smartphone’s camera picture via video call (in the living room); B=smartphone’s placement (in the living room); C= smartphone’s camera picture via video call (in the yard); D= smartphone’s placement (in the yard). Red marks on B and D show the smartphone and the used object (a pitcher and a mug in a table (elevated position) in the living room, and the patient’s shoe in the yard).


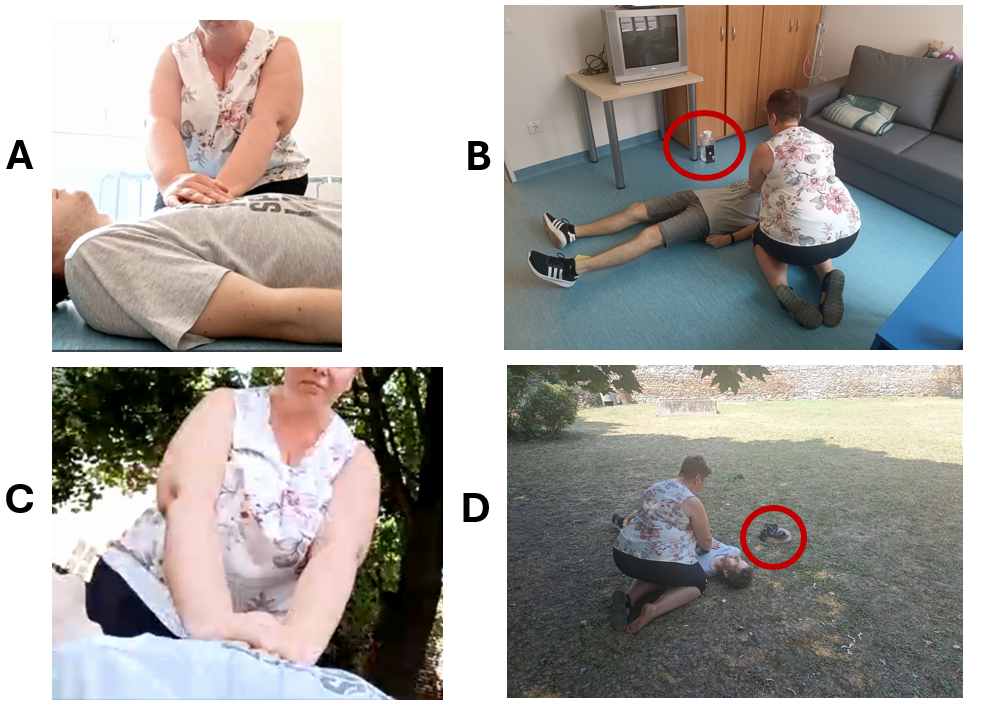
 **Fig. B.4.** Results of participant No.4. A=smartphone’s camera picture via video call (in the living room); B=smartphone’s placement (in the living room); C= smartphone’s camera picture via video call (in the yard); D= smartphone’s placement (in the yard). Red marks on B and D show the smartphone and the used object (a pitcher in the living room, and a flower holder and the lay responder’s shoe in the yard).


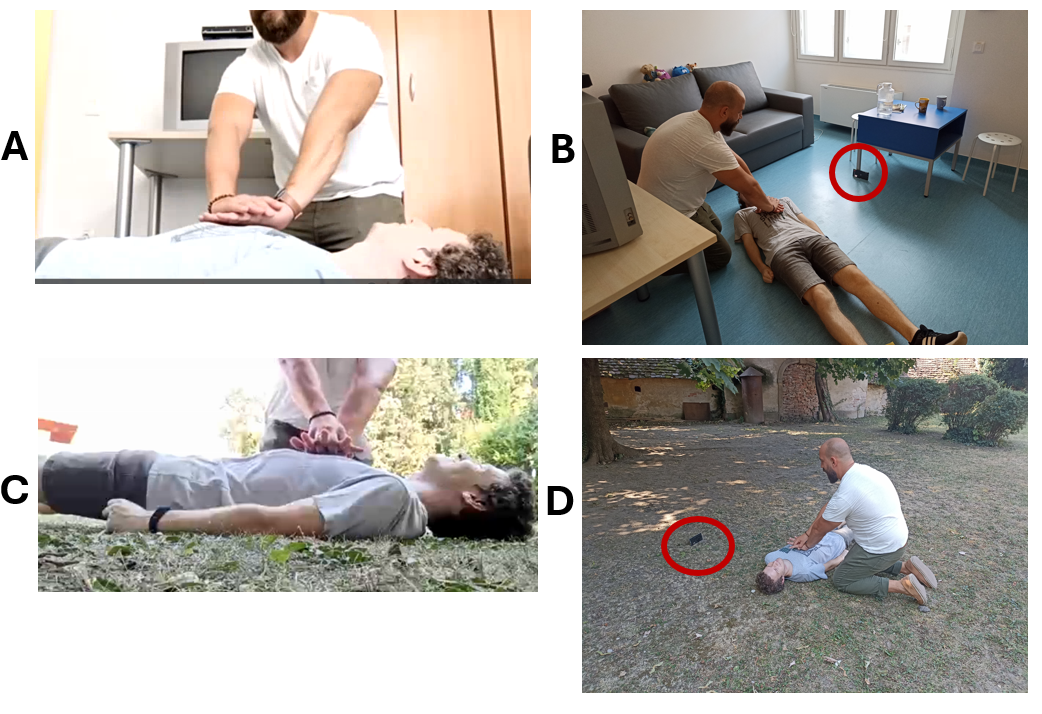
 **Fig. B.5.** Results of participant No.5. A=smartphone’s camera picture via video call (in the living room); B=smartphone’s placement (in the living room); C= smartphone’s camera picture via video call (in the yard); D= smartphone’s placement (in the yard). Red marks on B and D show the smartphone and the used object (a table leg in the living room, and a stone in the yard).


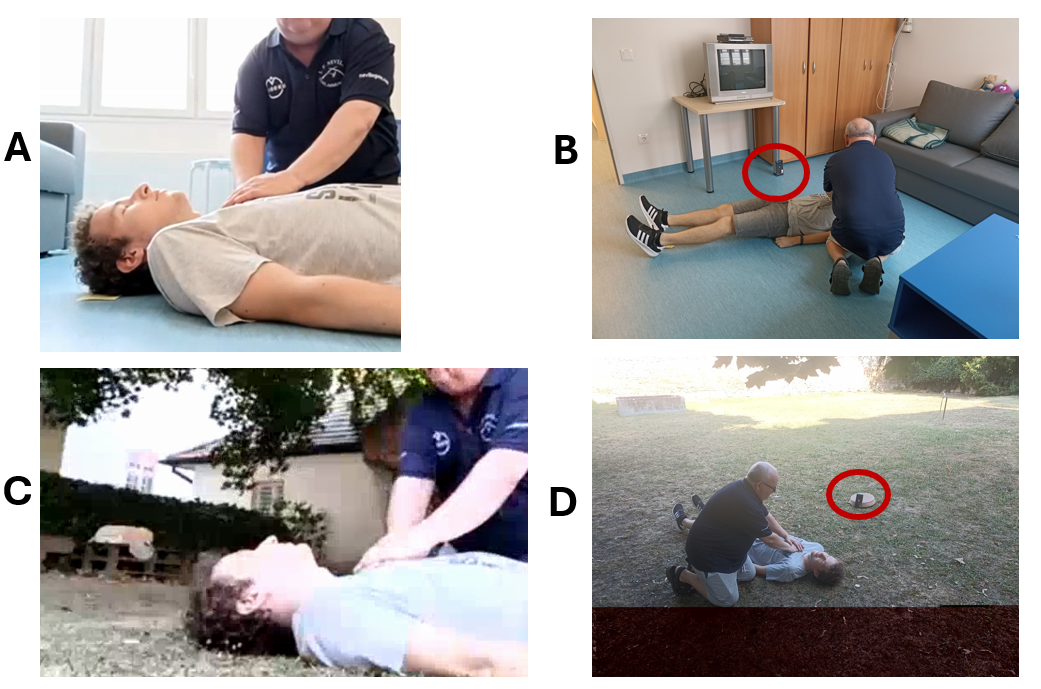


**Fig. B.6.** Results of participant No.6. A=smartphone’s camera picture via video call (in the living room); B=smartphone’s placement (in the living room); C= smartphone’s camera picture via video call (in the yard); D= smartphone’s placement (in the yard). Red marks on B and D show the smartphone and the used object (a table leg in the living room, and flower holder in the yard).


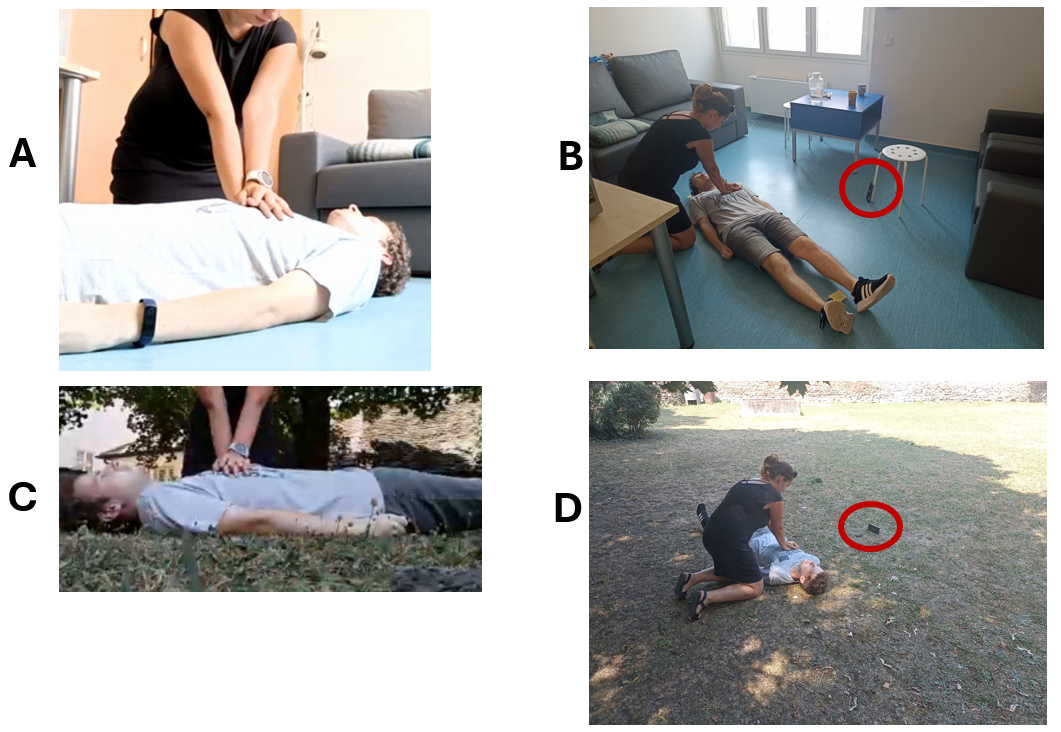


**Fig. B.7.** Results of participant No.7. A=smartphone’s camera picture via video call (in the living room); B=smartphone’s placement (in the living room); C= smartphone’s camera picture via video call (in the yard); D= smartphone’s placement (in the yard). Red marks on B and D show the smartphone and the used object (a chair in the living room, and stone in the yard).


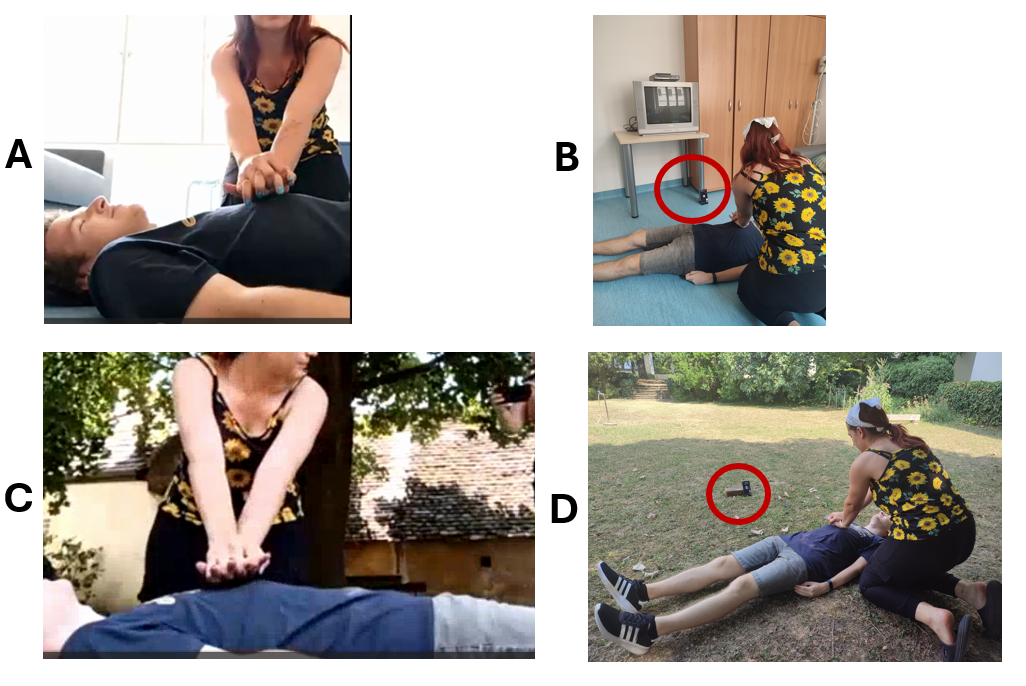
 **Fig. B.8.** Results of participant No.8. A=smartphone’s camera picture via video call (in the living room); B=smartphone’s placement (in the living room); C= smartphone’s camera picture via video call (in the yard); D= smartphone’s placement (in the yard). Red marks on B and D show the smartphone and the used object (a table leg in the living room, and brick in the yard).


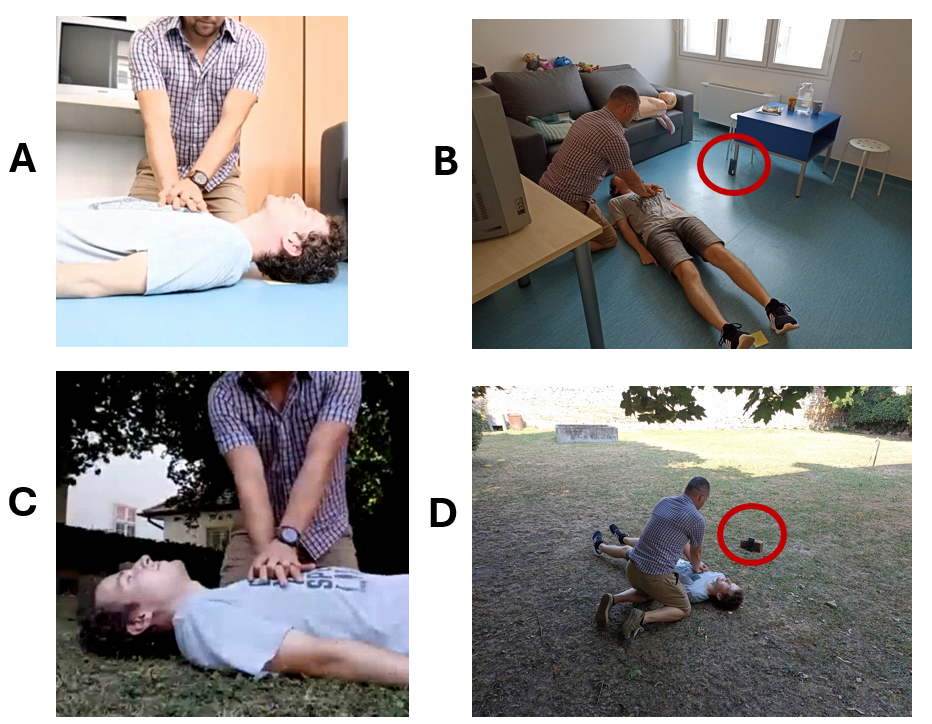


**Fig. B.9.** Results of participant No.9. A=smartphone’s camera picture via video call (in the living room); B=smartphone’s placement (in the living room); C= smartphone’s camera picture via video call (in the yard); D= smartphone’s placement (in the yard). Red marks on B and D show the smartphone and the used object (a table leg in the living room, and brick in the yard).


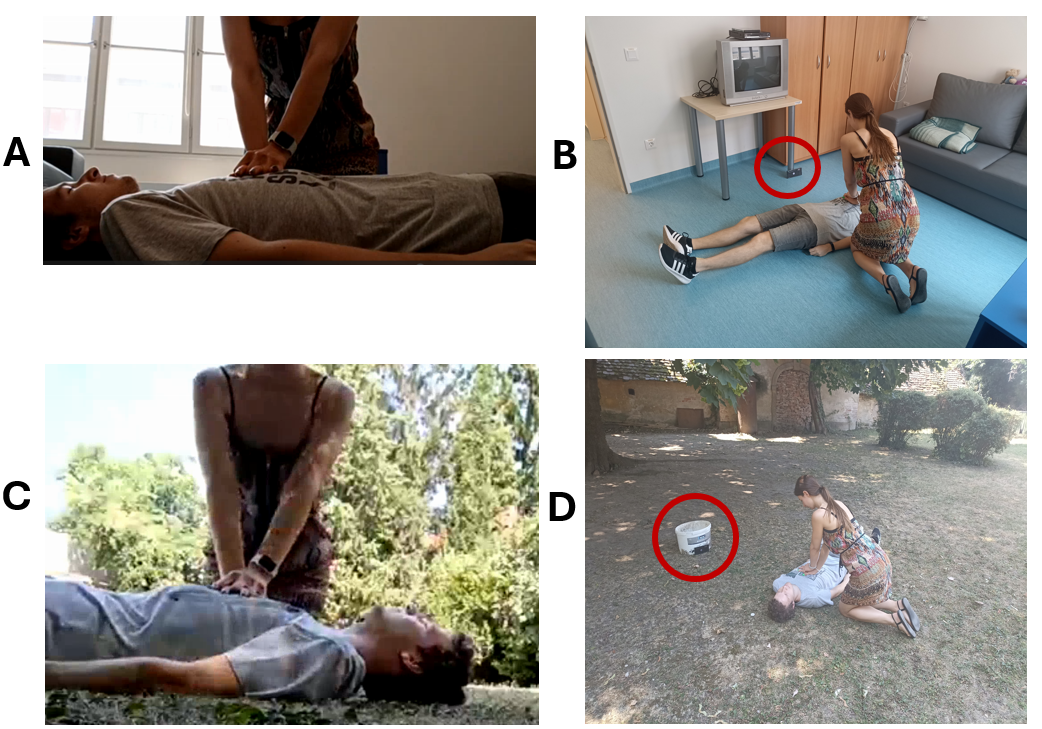
 **Fig. B.10.** Results of participant No.10. A=smartphone’s camera picture via video call (in the living room); B=smartphone’s placement (in the living room); C= smartphone’s camera picture via video call (in the yard); D= smartphone’s placement (in the yard). Red marks on B and D show the smartphone and the used object (a table leg in the living room, and a bucket in the yard).

**Supplementary Material 3: Subgroup comparisons** (location, smartphone placement, smartphone position, lighting, used object)

|  |  | **Overall** | **Location** | | | **Smartphone placement** | | | **Smartphone position** | | | **Lighting** | | | **Used object** | | |
| --- | --- | --- | --- | --- | --- | --- | --- | --- | --- | --- | --- | --- | --- | --- | --- | --- | --- |
|  |  |  | Living room | Yard | p-value | Ground | Elevated | p-value | Portrait | Landscape | p-value | Facing the light | Back to the light | p-value | Moveable | Fixed | p-value |
| **Assessability** | Hand position, mean (SD) ^b^ | 3.6 (0.5) | 3.9 (0.1) | 3.2 (0.6) | 0.01 | 3.6 (0.6) | 3.8 (0.0) | 0.858 | 3.7 (0.3) | 3.0 (1.1) | 0.283 | 3.5 (0.9) | 3.7 (0.2) | 0.511 | 3.5 (0.6) | 3.9 (0.1) | 0.079 |
|  | Hand placement, mean (SD)^a^ | 2.9 (0.6) | 3.4 (0.3) | 2.4 (0.4) | 0.013 | 2.8 (0.6) | 3.8 (0.0) | 0.141 | 3.0 (0.6) | 2.7 (0.7) | 0.561 | 3.1 (0.6) | 2.9 (0.7) | 0.665 | 2.7 (0.6) | 3.4 (0.2) | 0.119 |
|  | CC depth, mean (SD) ^a^ | 3.1 (0.8) | 3.6 (0.3) | 2.6 (0.7) | 0.019 | 3.1 (0.8) | 3.2 (0.0) | 0.919 | 3.1 (0.8) | 3.3 (0.7) | 0.732 | 3.6 (0.5) | 2.8 (0.8) | 0.154 | 2.8 (0.8) | 3.7 (0.1) | 0.095 |
|  | CC rate, mean (SD) ^b^ | 3.6 (0.9) | 4.0 (0.0) | 3.1 (1.1) | 0.013 | 3.5 (0.9) | 4.0 (0.0) | 0.433 | 3.6 (0.9) | 3.6 (0.6) | 0.659 | 3.8 (0.4) | 3.4 (1.1) | 0.548 | 3.4 (1.0) | 4.0 (0.0) | 0.123 |
| **Video quality** | Clarity, mean (SD) ^a^ | 2.8 (0.8) | 3.4 (0.6) | 2.2 (0.6) | 0.018 | 2.7 (0.8) | 3.4 (0.0) | 0.471 | 2.8 (0.8) | 2.8 (1.4) | 1.000 | 3.2 (0.9) | 2.5 (0.7) | 0.225 | 2.6 (0.8) | 3.3 (0.8) | 0.191 |
|  | Lagging, mean (SD) ^a^ | 2.9 (0.8) | 3.5 (0.2) | 2.4 (0.8) | 0.014 | 2.8 (0.8) | 3.8 (0.0) | 0.296 | 2.9 (0.9) | 3.1 (0.7) | 0.778 | 3.3 (0.5) | 2.7 (0.9) | 0.283 | 2.7 (0.9) | 3.5 (0.2) | 0.201 |
|  | Camera perspective, mean (SD) ^a^ | 2.9 (0.6) | 3.4 (0.2) | 2.4 (0.4) | 0.002 | 2.8 (0.6) | 3.6 (0.0) | 0.256 | 2.9 (0.7) | 2.9 (0.4) | 1.000 | 3.2 (0.4) | 2.7 (0.7) | 0.231 | 2.7 (0.7) | 3.3 (0.2) | 0.158 |
|  | General quality, mean (SD) ^a^ | 2.7 (0.8) | 3.3 (0.3) | 2.1 (0.5) | 0.002 | 2.6 (0.7) | 3.6 (0.0) | 0.241 | 2.7 (0.8) | 2.7 (0.9) | 1.000 | 2.9 (0.7) | 2.6 (0.9) | 0.536 | 2.5 (0.8) | 3.2 (0.3) | 0.196 |
| **Correctness of the assessment** | Hand position, n (%)^c^ | 86 (86) | 96 (96) | 76 (76) | 0.098 | 86 (86) | 80 (80) | 0.546 | 88 (88) | 80 (80) | 0.656 | 82 (82) | 4.2 (100) | 0.319 | 80 (80) | 100 (100) | 0.087 |
|  | Hand placement, n (%)^c^ | 78 (78) | 92 (92) | 44 (44) | 0.001 | 64 (64) | 100 (100) | 0.131 | 76 (76) | 90 (90) | 0.454 | 72 (72) | 50 (50) | 0.172 | 94 (94) | 58 (58) | 0.019 |
|  | CC depth, n (%)^c^ | 32 (32) | 32 (32) | 32 (32) | 1.000 | 32 (32) | 20 (20) | 0.482 | 24 (24) | 60 (60) | 0.123 | 18 (35.0) | 20 (20) | 0.468 | 26 (26) | 46 (46) | 0.146 |
|  | CC rate, n (%)^c^ | 84 (84) | 84 (84) | 84 (84) | 1.000 | 84 (84) | 80 (80) | 0.599 | 86 (86) | 80 (80) | 0.653 | 40 (80.0) | 100 (100) | 0.184 | 82 (82) | 86 (86) | 0.549 |
| **Time of placement** | Mean time, sec (SD) ^b^ | 28.7 (16.6) | 23.4 (16.6) | 34.0 (16.6) | 0.075 | 30.1 (16.9) | 40 (0.0) | 0.295 | 31.3 (17.6) | 18.5 (6.3) | 0.295 | 17.8 (3.9) | 36.0 (18.1) | 0.069 | 28.9 (16.1) | 28.3 (21.5) | 0.568 |

Table C.1. Subgroup analysis of the assessors' results based on the location, smartphone position, smartphone placement, lighting and the used object. Video quality scores were given using a 4-point Likert scale: 1 – very bad, 2 – bad, 3 – good, 4 – very good. The assessability of the chest compressions was evaluated using a 4-point Likert scale: 1 – very bad, 2 – bad, 3 – good, 4 – very good. The correctness of the evaluation was calculated based on the prior evaluation of the authors and the QCPR software. In the „Correctness of the assessment category,” results show how much time (n, %) was assessment correct from 100 video views (5 assessors / 20 videos = 100 views, in total), in every CC parameter (hand position, hand placement, depth, rate).

CC= chest compression, SD= standard deviation.

^a^ t-test was used (data are normally distributed)

^b^ Mann-Whitney U-test was used (data are not normally distributed)

^c^ Chi-square-test was used
